# The Canadian Natural Health Products (NHP) regulations: industry perceptions and compliance factors

**DOI:** 10.1186/1472-6963-6-63

**Published:** 2006-05-31

**Authors:** Hina Laeeque, Heather Boon, Natasha Kachan, Jillian Clare Cohen, Joseph D'Cruz

**Affiliations:** 1 Leslie Dan Faculty of Pharmacy, University of Toronto, Toronto, Canada; 2 Department of Health Policy, Management and Evaluation, Faculty of Medicine, University of Toronto, Toronto, Canada; 3 Department of Family and Community Medicine, Faculty of Medicine, University of Toronto, Toronto, Canada; 4 Rotman School of Management, University of Toronto, Toronto, Canada

## Abstract

**Background:**

The use of natural health products, such as vitamins, minerals, and herbs, by Canadians has been increasing with time. As a result of consumer concern about the quality of these products, the Canadian Department of Health created the Natural Health Products (NHP) Regulations. The new Canadian regulations raise questions about whether and how the NHP industry will be able to comply and what impact they will have on market structure. The objectives of this study were to explore who in the interview sample is complying with Canada's new NHP Regulations (i.e., submitted product licensing applications on time); and explore the factors that affect regulatory compliance.

**Methods:**

Twenty key informant interviews were conducted with employees of the NHP industry. The structured interviews focused on the level of satisfaction with the Regulations and perceptions of compliance and non-compliance. Interviews were tape recorded and then transcribed verbatim. Data were independently coded, using qualitative content analysis. Team meetings were held after every three to four interviews to discuss emerging themes.

**Results:**

The major finding of this study is that most (17 out of 20) companies interviewed were beginning to comply with the new regulatory regime. The factors that contribute to likelihood of regulatory compliance were: perceptions and knowledge of the regulations and business size.

**Conclusion:**

The Canadian case can be instructive for other countries seeking to implement regulatory standards for natural health products. An unintended consequence of the Canadian NHP regulations may be the exit of smaller firms, leading to industry consolidation.

## Background

Consumer use of vitamins, minerals, and herbs increased dramatically during the 1990s. A 2001 survey found that 75% of Canadians had used a natural health product (NHP), such as vitamins, minerals, and/or herbal remedies, for a health condition[1]. In the United States, the use of herbals increased 380% during the period of 1990 to 1997[2]. As the use of these products increased, so did concern among consumers over the quality and efficacy of NHPs.

The Canadian federal department of health, Health Canada, conducted extensive consultations with industry members, consumers, health care providers, and researchers to assist in developing regulations specifically for NHPs. Health Canada began implementing the NHP regulations on January 1, 2004[3]. The new NHP regulations classify NHPs as a sub-category of drugs, and apply to products commonly known as traditional herbal remedies, traditional Chinese, Ayurvedic, and Native North American medicines, homeopathic medicines, and vitamin and mineral supplements. Health Canada seeks "to ensure that all Canadians have ready access to natural health products that are safe, effective, and of high quality, while respecting freedom of choice and philosophical and cultural diversity"[4]. The regulations specify industry requirements for selling NHPs in Canada, including manufacturing, packaging, labelling, storing, importing, and distributing specifications.

Prior to January 2004, NHPs were officially regulated pursuant to the *Food and Drugs Act *(1985), mainly depending on whether medicinal claims were made. However, many companies did not follow either the food or drug regulations and neither were rigorously enforced. The NHP regulations are viewed as being more strict than the food product regulations because companies must provide evidence of safety, efficacy, and quality to Health Canada, but less strict than the regulation of drugs in the country. The regulations represent government monitoring over the sale of NHPs via pre-market approval mechanisms.

Current debate is focused on what impact these regulations will have on the industry and how once their impact is known, this will shape the regulations. Very little is known about how the industry will respond to the NHP Regulations. This paper hopes to fill in some of the gaps by examining how industry is responding the the regulations.

### II. Background

A number of factors that affect compliance with new regulations have been identified in the literature including: perceptions of the law (whether the law is thought to be necessary, whether those having to comply with the regulations agree with the law and feel they would benefit from the law), the perceptions of the regulated companies towards compliance with the law (whether noncompliance will result in penalties, whether the type and amount of compliance is clearly stated in the law, and whether compliance can be supported), as well as whether the law specifies enforcement activity and the regulated respond to the law[5]. Compliance of those being regulated has been identified as an important stage of policy implementation and achieving the goals of a regulation[6]. Thus, this study investigates whether defined factors for individual compliance behavior are applicable to industry compliance with the new NHP Regulations. Before detailing the findings of the study, additional information about the NHP Regulations and the NHP industry is provided for context.

#### A. The Natural Health Products (NHP) regulations

On of January 1, 2004, the NHP regulations became law administered by the Natural Health Products Directorate (NHPD) of Health Canada. Any company that sells a finished form of a NHP on the Canadian market must submit a product licence application (PLA) to the NHPD. The PLA must contain evidence for the safety, efficacy, and quality of the product. For example, specific baseline studies are required (unless the product already has a significant history of safe use in humans) such as repeat dose toxicity studies, genotoxicity studies and reproductive toxicity studies which are performed according to international standards such as the World Health Organization, International Conference on Harmonization and/or the Organization for Economic Cooperation and Development[4]. The application must also include information supporting the health claim of the NHP. An eight-digit natural product number (NPN) or a Drug Identification Number-Homeopathic Medicine (DIN-HM) (for homeopathic medicines) is issued by the NHPD to the company upon review and acceptance of the provided evidence. According to the NHP Regulations (2003), the NPN or DIN-HM must be displayed on the product label when sold on the Canadian market as indication of market authorization. All Canadian products which were on the market on January 1, 2004 (when the new regulations became law) have up to six years to obtain product licences. However, products new to the market after that date require pre-approval prior to sale. The Canadian NHP market is estimated to include up to 50,000 individual products, all of which must submit product licensing applications (PLAs) to NHPD. This has created a substantial backlog and as of January 2005 there were thousands of application in cue waiting for NHPD assessment.

During the period of 2004 to 2010, Health Canada decided to focus its activities by assigning all NHPs to six "priority" categories based loosely on perceived risks associated with the products in each category. Each priority category has its own deadline for submitting PLAs in an attempt to ensure that they arrive at NHPD throughout the six year transition period. For example, Priority One products are products meeting the definition of a natural health product listed on the Therapeutic Products Directorate's (TPD's) *Listing of Drugs Currently Regulated as New Drugs*[7] and must comply with the NHP regulations by June 30, 2004. Examples include glucosamine, and some forms of devil's claw, dong quai, and ginseng[8]. That is, companies that sell a product on this list must have applied for a NPN by June 30, 2004. After this date, the company will be subject to enforcement actions by the Health Products and Food Branch Inspectorate (HPFBI), such as voluntary disposal, voluntary detention, recall, warning, stop sale or prosecution[9]. Any company that wishes to bring a new product to market whose ingredients fall within the priority one category must receive pre-approval prior to selling the new product in Canada.

The substances in this category are high priority because Health Canada claims that little information exists on the safety and efficacy of the use of these products for medicinal purposes. Companies that sell chondroitin and glucosamine (Priority One products) in Canada were specifically targeted to participate in this study. Chondroitin and glucosamine products were chosen because: all forms for all indications are clearly listed as Priority One products (i.e., must meet the June 30^th ^deadline); there are many different brands currently available on the Canadian market; and a significant amount of scientific evidence supporting the efficacy of both chondroitin and glucosamine in the management of osteoarthritis is available. Thus, industry members may be eager to register these products in order to make health claims on their packaging and promotion.

#### B. Overview of the NHP industry in Canada

Information on the NHP industry in Canada is limited because NHPs are not part of the established health care system in the country; neither the industry nor the government monitor NHP sales as a distinct category, and provincial drug plans do not cover purchases of NHPs[1]. According to a business impact test (BIT) (2003) conducted prior to the implementation of the Regulations for Health Canada, the NHP industry is composed of mainly small and cottage businesses. Fifty-five percent of respondents in the BIT identified themselves as part of a company with fewer than 19 employees and 75% of respondents said their company had fewer than 50 employees. These percentages from the survey are said to be indicative of the NHP industry in Canada.

The purpose of this paper is twofold: first, to explore who in the study sample is complying with the Canadian NHP Regulations (i.e., submitted product licensing applications on time); and second, to investigate factors that affect regulatory compliance.

## Methods

The study is an applied ethnography[10] that incorporates qualitative research methods in order to better understand the perceptions of NHP industry members in Canada. This study explores the factors of firm compliance in several novel ways. First, the study utilizes an ethnographic approach that uses qualitative methods. Qualitative methods, such as interviews, are particularly useful in understanding complex social processes, such as the implementation of federal regulations. Interviews are useful for understanding human perception and behavior, which is difficult to obtain from survey data. Second, the study was conducted during the very early stage of implementation, rather than after full implementation. Third, the study does not assume that the factors that affect compliance will be the same for different business sizes and activities.

### Interview sample

Criterion-based, purposeful sampling[11] was used to select NHP companies that are required to submit applications for NPNs. To be included in the study, a company had to meet the following criteria: a) it sells finished forms of chondroitin and/or glucosamine on the Canadian market; b) it is required, according to the NHP Regulations, to submit PLAs for glucosamine and/or chondroitin by the June 30, 2004, deadline; c) it has an office located in Canada; and d) representatives are able to participate in an interview conducted in English. There were no exclusion criteria. Of the approximately 364 NHP businesses in Canada, 67 met the eligibility criteria of the study. From the 67 companies, companies were selective chosen to ensure a maximum variation sample[11] with respect to business activity/specialty, size, and location.

### Data gathering

Semi-structured interviews were conducted either by telephone or in-person. The person responsible for regulatory affairs and/or writing the NPN applications was interviewed in each case. The interviews were scheduled for approximately one hour and were audio taped and transcribed verbatim. Prior to the interview, each respondent was mailed a survey to collect basic information about the size of the company, number of NHPs they sell and details about the glucosamine/chondroitin products they sell. At the end of each interview, the interviewer reviewed the survey with the respondent in an attempt to ensure that all questions were answered accurately and fully. These questions remained fixed through out the study. See Additional file 1 for interview guide and survey questions.

The interviews focused on the perceptions of individuals regarding the regulations, such as: whether the regulations are necessary and whether they agree with the goal of the regulations. The respondents were also asked about the likelihood of regulatory enforcement and industry compliance. The interview questions were developed based on information from the BIT study, consensus among the research team, and responses from participants early on in the study. The specific interview questions changed as the research proceeded in order to follow-up on information and themes identified by participants as important. Interviews continued until a point of saturation was reached. Saturation is the point in qualitative research where no new information about key themes is emerging from new interviews [11].

### Company representatives interviewed

**Figure 1:**
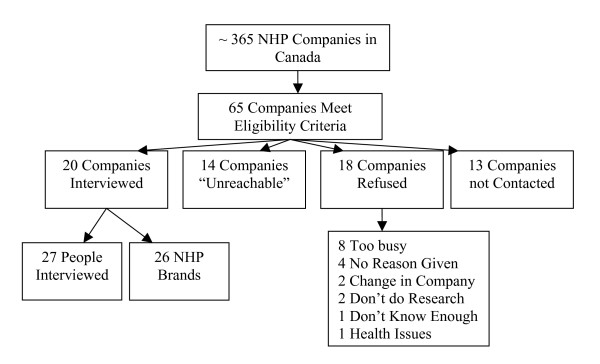
Companies Interviewed for the Study.

The president/owner, regulatory affairs manager, and/or quality assurance personnel were interviewed at 20 of the 67 eligible companies. Thirteen companies were considered "unreachable" after leaving three voice messages for the contact person at the company and 18 declined to participate. The remaining 13 companies were not contacted because saturation[11] had been reached after conducting 20 interviews (Figure 1).

NHP companies of various locations, specialties, and sizes were included (Table 1). The majority of companies that were involved in the study are based in Ontario (11 companies), British Columbia (3 companies), and Quebec (3 companies). The remaining companies are located in Alberta, Manitoba, or had head offices in the United States. The different NHP specialties include manufacturing (18 companies), distributing (11 companies), private labelling (5 companies), formulating (4 companies), as well as many others. These companies sell their products in health food stores, large distribution chains, the internet, and through health practitioners. Lastly, small, medium, and large sized companies were involved in the study.

Based on the BIT definition of business size, 9 companies in the study are large, 6 are medium, and 5 are small businesses (Table 1). The majority of companies in the study had annual sales of less than 10 million dollars, sold several hundred NHP products and less than 100 DIN products on the Canadian market, began operations post 1991, and primarily focused product sales in Canada. The study also captures companies in the extreme positions of the NHP industry. For example, companies that have as little as three to as high as 500 employees were involved in the study as well as sales ranging from half a million to over 200 million dollars. Given this, a range of perceptions can be described with confidence.

### Data analysis

Data collected from the interviews were independently analyzed, using qualitative content analysis[12], by three different researchers (HL, HB, and NK). Content analysis involves analyzing the interview transcripts by categorizing segments of the transcripts into topic areas[13,14] Transcript segments can vary in size from a single word to paragraphs. In the analysis, the were transcripts into simple sentences with subjects and predicates, called "themes"[13]. Each theme was then placed in a topic category based on its content. These themes cannot be identified *a priori*, but rather must emerge from the data collected. Large categories were further divided into sub-categories creating a tree-diagram. Research team meetings were held after every three interviews to discuss the coding process and key themes emerging from the data. The qualitative analysis software NVIVO[15] was used to store and manage the data collected from interviews.

The perceptions of individuals from small and medium companies have been combined and are referred to as small and medium sized enterprises (SMEs), given the similarity of the issues identified from their interviews. The opinions of representatives of SMEs are compared and contrasted to those of large firm representatives. These opinions are portrayed via direct quotations from interview participants.

## Results

### A. Business size and compliance with the Priority One products deadline

In this study, compliance is measured by whether a company submitted a product licence application to the NHPD for chondroitin and/or glucosamine by the June 30, 2004, deadline. For the study sample, compliance with the deadline varied according to business size (Table 2). For example, 3 SMEs were noncompliant with the deadline (failed to submit a PLA by the deadline date), 4 SMEs were under complying (submitted a PLA after the deadline date), 1 SME was semi-complying (some of company PLAs submitted by the deadline), and 3 SMEs were compliant. In comparison, no large companies were noncompliant or under complying, 5 were semi-complying, and 4 were compliant. Important to note is that only SMEs fall into the noncompliant and under compliant category. Since compliance with the first NHPD deadline varies with business size, the factors that affect compliance are considered with respect to business size.

### B. Perceptions of the NHP regulations

#### Whether the regulations are necessary

All employees interviewed agree with the goal of the NHPD, that is, to ensure the safety, quality, and efficacy of NHPs. The majority of participants felt that the regulations are necessary for reasons such as establishing industry standards and increasing consumer confidence in NHPs. Many participants also stated that the regulations would ensure a fair, level playing field. That is, any firm that produces a NHP must abide by the NHP regulations, and no longer has the option of complying with either the food or drug standards.

#### Level of agreement with the NHP regulations

In general, participants agree with the intent of regulations although the intensity of agreement varied with business size.

#### Large firms

Overall, employees of large firms perceive the regulations more positively than employees of SMEs. The majority of participants from large firms stated they were satisfied with the final version of the regulations in comparison to participants from SMEs. An individual stated that the regulations were well written and that the NHPD had consulted with other Directorates in Health Canada in order to develop practical regulations.

On the other hand, some representatives from large companies are disappointed with the time frames provided by the NHPD but for different reasons than representatives from SMEs. Large firm employees are frustrated that the implementation stage is moving slowly:

the faster the weaker players get out of the business, the better, from a commercial side or predatory side ... there's only going to be 3 or 4 big players that are going to survive. We intend on being one of them. So I'm saying if you're going to cause that, then do it faster. Quit slow bleeding us. (6, Large)

Many feel that the regulations will increase the public's confidence in the NHP products and thus benefit the NHP industry in the long run, but the process of getting to that position is painful:

I think it's guaranteeing the safety of the Canadian public, and standardizing the industry...So in the long run, it's going to be better for us. It's just going to be painful getting there (6, Large).

#### SMEs

Several SME participants did not agree with the way the regulations were being implemented. For example, many participants stated that the regulations are too strict for the level of risk NHPs pose to the general public. A representative from a SME said "I think that Health Canada is being too safe with the products that have a long history of use on safety...products that have been sold for so long with very few adverse events being reported" (13, Medium). Others expressed similar concerns:

No, I'm not [satisfied]. The major reason is I think it [the NHP Regulations] is much too onerous for ... the level of risk we pose to the general public... the vast majority of whatever is being produced in the natural product industry does not come anywhere close to drugs as far as the safety issues are concerned (5, Small).

SMEs representatives are the most dissatisfied with the NHP Regulations because they feel the regulations impose the same level of control as the drug regulations in Canada. They feel that individuals who wrote the regulations had experience in the pharmaceutical industry and very little experience in the NHP industry. The regulations are described as impractical for small NHP businesses. One participant stated, " [the NHP regulations] just lack reasonable expectations for business. It [the NHPD] approached the business as if we're all large drug manufacturers with millions of dollars of research and patents" (7, Small). Similarly, many participants do not like that the government considers NHPs a sub-set of drugs because NHPs do not have the volume of sale, price mark-up, and level of risk associated with pharmaceutical drugs.

As a result, many participants felt that small businesses with few products would not be able to survive in the new regulatory environment. For example, a key informant stated:

I've got roughly a hundred products...how many am I going to lose? I would say I'm going to lose probably one to two out of five products...will not meet the requirements of the Regulations...the fact there are no human clinical trials done on those ingredients (7, Small)

Several key informants from SMEs stated that smaller businesses in the industry produce niche products and large firm produce standard vitamins and minerals. Since smaller companies may not be able to afford to market entire product lines, their innovative products may be lost from the industry.

Another concern was the compliance timelines set out by the NHPD. Small companies felt the timelines were too short, especially those associated with the priority phase-in strategy. There was also frustration expressed about what was perceived as the changeable nature of what was required and when:

I'm also concerned that their timelines are so tight. You know, initially the priority one applications were supposed to be in by June 1^st^, they extended it to June 30^th^, and in June they put out that document saying, you know, "if you submit an application before June 30^th^, then you can sell the product. If you submit it after, you can't sell it until you get your approval. (8, Medium)

### C. Perceptions of compliance

#### Large firms

Only employees of large companies are concerned that other firms in the NHP industry will fail to comply with the new regulations. One participant stated that 99% of his contacts are not changing their operating procedures because they believe that the regulations are not going to last. Another participant voiced a similar concern stating that the backlog in product review is, in essence, preventing companies from launching novel products on the Canadian market. As a result, some may disregard them in order to survive as a company. Another said, "the tricky part right now is to get everybody to buy into them [the NHP regulations] and say, 'yes, we are operating in the industry, these are the regulations we are going to abide by and we all support it.' Like I said, the biggest danger to these new regulations is mass non-compliance." (4a, Large)

#### Enforcement and noncompliance

##### Large firms

Many participants feel that very few regulatory enforcement actions are currently taking place. One person stated, "they're [government officials] not doing anything." (4b, Large) Many suggested little enforcement action is occurring because of the large number of PLAs the NHPD is currently processing and limited resources of the government: "the HPFBI, the Inspectorate, has already indicated they don't have the resources to be policing this, so the reality is they are going to rely on people snitching, and the industry has not typically snitched on each other." (9, large) One individual felt that the enforcement actions would take place once the backlog disappears, in approximately five years.

Several participants were concerned about how the regulations will be enforced in the future. Many are concerned that compliance actions will be too strict, will focus on only part of the NHP industry, and will be reactive rather than proactive:

I think that they are going to enforce ... I can see probably the companies that are spending the most and doing the most are the ones that are going to be targeted. Because they're also the higher profile ones. And that's how they implement and how they enforce. (6, Large)

##### SMEs

Representative from SMEs were more certain that the regulations would be strictly enforced. A representative of a small company stated that he was disappointed that people are talking about enforcement issues. Instead, he would like to see more discussion around how the government can provide assistance to companies during the transition period:

The bottom line is, are we really to that point where we have to talk about enforcement of companies that have been in business for the better part of ten or twelve years? ... [the federal government should say] "There's going to be a transition period and here's how we're going to help you meet that. We're going to help you by coming out and going through your place and telling what you need to do...We'll come in to help you."(7, Small)

### D. Other factors that affect compliance

#### Knowledge of the regulations

##### Large firms

Representatives from large firms were very knowledgeable about details of the regulations, including how the regulations were formed and the details and requirements in the regulations. Many participants stated that the company has either vitamin or mineral products with a Drug Identification Number (DIN) and thus are compliant with the drug regulations under the *Food and Drugs Act*, of which the NHP regulations are perceived to be a less strict version. A participant stated, "a lot of it [the NHP regulations] is common sense. It's having the people together, that information that you already have within your company, putting it into a standard format, and submitting it [to the NHPD]" (4a, Large). This individual continued by explaining:

They [the NHPD] loosened up on their manufacturing standards...where they tightened up was pre-product approval. So the activities and the amount of information needed to get pre-market approval of NHPs have increased extremely. But I think that is in compensation because the manufacturing standards were lowered. (4a, Large)

##### SMEs

This group of companies revealed the most misinformation about the regulations compared to the other companies. For example, one participant thought Health Canada inspectors would be testing all NHPs on the Canadian market to ensure safety. Yet others were unaware of when and how the regulations would be implemented.

## Discussion

The major finding of this study is that 17 of 20 companies are in the process of submitting PLAs to the NHPD. The three noncompliant companies were SMEs, of which none knew that they had failed to comply with the deadline.

Determining whether perceptions of the regulations, including whether the law is necessary and the intensity of agreement with the regulations, is an important factor for compliance is difficult in this study because all participants felt the Regulations are necessary and agree with the mandate of the NHPD. Despite general agreement with the mandate, employees of SMEs were concerned about how rigorously the regulations would be applied and the potential negative impact on their businesses. In comparison, large firm employees were satisfied with the standards and looked forward to full implementation. Despite some misgivings expressed by some SMEs, the majority of the companies in the sample were submitting PLAs, which seemed to be at least in part because of their generally positive views of the regulations. Thus, perceptions of regulations are an important compliance factor.

This study also addressed perceptions of noncompliance and enforcement. Only representatives from SMEs were certain that noncompliance with the regulations would result in enforcement actions and indicated that this contributed to their compliance. In contrast, large firm representatives were confident that the regulations would not be enforced and were concerned that other firms would not comply with the regulations. Despite these beliefs, large firms were complying with the regulations. This means that neither the perception that noncompliance will result in penalties, nor the likelihood of other firms complying, affects the compliance of large firms.

The only factor that influences compliance for both large firms and SMEs is knowledge of the regulations. The acquired knowledge from complying with other government regulations lead to compliance with the NHP regulations for large firms, whereas the lack of knowledge of the NHP regulations leads to noncompliance for some SMEs.

The finding that those not complying were not avoiding the regulations, but rather that they were unaware of their responsibilities to comply, is a phenomenon that has also been shown by a study of self-reporting about chemical emissions by American manufacturers. Non-reporters were generally smaller firms that were ignorant of reporting requirements[16].

### VIII. Implications of the NHP regulations on the industry

#### The structure of the NHP industry

As explained in the introduction, very little is known about the Canadian NHP industry. Nevertheless, three key characteristics of the industry are known: that 50% of the NHP industry is comprised of small and medium sized business, that a few large, multi-million dollar firms have a significant portion of the market share, and that Canadian sales for the industry are approximately $4.3 billion. Participants of the study believed that larger companies offer commonly used NHPs, such as vitamins and minerals, and smaller firms produced more niche products for particular clientele, such as products with several active ingredients. With these characteristics in mind, the potential impact of the NHP regulations on the industry can be assessed.

#### The NHP regulations and SMEs

Since a significant number of NHP companies are SMEs and this paper explores the experiences of industry members with the new regulations, predictions can be made about the impact of the regulations on SMEs. A finding of this study is that three firms were not complying with them: all three firms were SMEs. These three SMEs were not deliberately avoiding regulatory compliance. Rather, employees and/or owners had little to no knowledge of the regulations.

In addition, employees of smaller firms that were complying expressed concern about how their firms would survive in the new regulatory environment. In particular, participants were frustrated with how the regulations are being implemented. Furthermore, smaller firms are also less likely to register products by the appropriate deadline. Thus, some SMEs are having difficulties in complying with the product licensing requirements.

These findings suggest that a possible impact is that some small firms may not be able to survive the exigencies of the regulations and close their businesses. Thus, the NHP industry may become more concentrated as a way to ensure economies of scale. That is, the current industry with 50% of SMEs and few large firms with a large market share may change to an industry with fewer small firms and large firms having an even greater market share than the present time. Thus, consolidation of the NHP industry is likely. If smaller firms are forced to exit the industry, some may choose to amalgamate with larger firms.

#### Exit of smaller firms from the industry

If firms that cannot afford to comply with the regulations are forced to exit the market, consumers may be protected if marginal firms and products disappeared and that might lead to greater consumer confidence in the use and safety of NHPs. These are the potential positive changes that the NHPD suggested would occur when the regulations were implemented.

On the other hand, it is by no means clear that the small firms that may exit the industry are currently making inferior products. Participants in this study seemed to think that smaller firms generally offer specialty products which certain consumers demand. If these smaller firms are forced to exit the industry, then many of these specialty products may no longer be available to consumers. This is particularly disconcerting, because one role of the NHPD is to allow for safe, high quality products without restricting consumer access to NHPs.

The question then becomes, what will consumers do without the products they have enjoyed for years? A potential outcome is a growth in the unregulated market of NHPs. So, products that are no longer legally available in Canada could be sold to consumers through unregulated channels, thus leaving consumers open to potential harm. Although illegal, some retail stores or health practitioners may be willing to provide products that are no longer available legally in the country. Internet sales of products imported from other countries for personal use may increase in response. Overall, the exit of some firms and loss of products will cause concern for some, but perhaps few, consumers. This is an area where further research is needed.

#### Consolidation of the industry

SME representatives were concerned that the NHP regulations would make it difficult for small firms to survive. Participants suggested that industry consolidation could lead to less innovative products and practices in Canada because many believe that the SMEs are the main innovators in the industry. Participants suggested that less innovation might occur because NHPs often cannot be patented and thus historically firms have generally not invested significantly in research and development. Compliance costs may also decrease the likelihood of financial investment into innovation. In contrast, other components of the regulations (such as the Natural Health Products Research Program) and the ability to make label claims based on scientific research may actually encourage research and innovation. Whether regulations encourage or discourage innovation is a current policy debate[17]. It remains unclear how the NHP Regulations may affect innovation in Canada.

SME employees also suggested that the regulations might encourage large foreign companies to sell more of their products in Canada. If some companies exit the industry, there may be a demand for products that might be filled by American, Asian, and European companies, which have already captured 75% of the global nutritional product industry [18]. As suggested by this research, larger companies are more capable of processing a PLA and thus able to sell products with the new NPN. Canadian NHP producers may not welcome the growth of foreign investments in the industry, but consumers may enjoy the diversity of product lines.

Large firms representatives also predicted consolidation of the industry and viewed this potential change as a positive feature of the NHP regulations because fewer firms operating in the industry would offer greater market opportunities. In addition, large firms that are presently operating in the industry have the advantage of familiarizing themselves with the NHP Regulations during the early stages of implementation. Thus, the NHP Regulations appear to serve the incumbents. This means that Canadian entrepreneurs may encounter greater barriers for entry into the market, particularly for small business owners; however, those already in the industry would appear to have an advantage.

#### Study limitations

The main limitation of this study is that the companies were selected based on their sale of chondroitin and/or glucosamine. These companies may be different from the other companies in the industry due to the nature of products they sell. Also, a large number of companies declined to participate. However, a maximum variation sample and saturation were successfully attained in the study, and data collection was purposely stopped after 20 interviews were completed because it appeared that additional interviews would not have provided significant new information. Despite this, it is not possible to determine whether those companies that declined to participate are complying with the new regulations. Another limitation of the study is that the response to compliance deadlines was based on self-reporting. However, participants were generally candid during interviews, leaving no reason to believe the information was misrepresented. Finally, some interviews were conducted in person, while others were completed using the telephone creating the potential that different quality or types of data may be collected depending on the method used. However, analysis of the interviews did not reveal any differences.

## Conclusion

In conclusion, the majority of NHP companies in the study are complying with the NHP regulations to varying degrees. Participants agree with the goal of the NHPD and the need for regulations. Compliance with the Regulations is associated with firm size as well as knowledge of the Regulations. That is, large firms that are familiar with the set of laws are more likely to comply with the Regulations. These findings suggest that smaller firms may exit the industry, leading to industry consolidation in Canada.

## Abbreviations

BIT: business impact test

CAM: complementary and alternative medicine

DIN: drug identification number

DIN-HM: drug Identification number-homeopathic medicine

HPFBI: Health Products and Food Branch Inspectorate

NHP: natural health product

NHPs: natural health products

NHPD: Natural Health Product Directorate

NPN: natural product number

PLA: product licensing application

SME: small and medium enterprises

TPD: Therapeutic Products Directorate

## Competing interests

The author(s) declare that they have no competing interests.

## Authors' contributions

HL conducted the interviews in collaboration with NK, as well as completed field notes and drafted the original manuscript. HL, NK, and HB were involved in data analysis. HB conceived the study design, provided assistance with qualitative research methods, and guided the research process. JCC and JD provided input into the research process. All authors read, provided suggestions on and approved the final manuscript.

## Pre-publication history

The pre-publication history for this paper can be accessed here:



## Supplementary Material

Additional file 1 Appendix 1 (word document) provides the interview guide and standard questions asked of each interviewee. Click here for file

## Figures and Tables

**Table 1:** Key characteristics of the 20 interviewed companies.

**Description**	**# of Companies**
**Size**	
Small (< 19 employees)	5
Medium (20 to 49 employees)	6
Large (> 50 employees)	9

**Annual Sales (Can $)**	
< 10 M	8
5 to 50.9 M	5
> 50 M	4
Confidential	3

**# of Products**	
< 99	2
100–499	13
500–999	4
>1000	1

**Market Focus**	
Local	0
Regional	1
National	15
International	9

**Table 2:** Company Compliance Status for the Priority One compliance deadline of June 30, 2004.

**Description**	**Number of Businesses in each Category**
**Noncompliant **(did not submit a PLA by June 30, 2004)	3 SMEs

**Under Complying **(sent in applications after June 30, 2004)	4 SMEs

**Semi Compliant **(ie. Company has more than one product, some PLAs submitted before June 30, other PLAs submitted after the date)	1 SMEs 5 Large

**Compliant **(submitted PLA by June 30, 2004)	3 SMEs 4 Large

**TOTAL**	20
