# Supplementary material for: The Canadian Natural Health Products (NHP) regulations: industry perceptions and compliance factors
Source: BMC Health Serv Res. 2006 May 31;6:63. doi: 10.1186/1472-6963-6-63 (PMC1524757; doi:10.1186/1472-6963-6-63)
Supplement: Additional file 1 — Appendix 1 (word document) provides the interview guide and standard questions asked of each interviewee. [file 1472-6963-6-63-S1.doc]

**Appendix A: Interview Guide**

1. Can you describe your company to me?

Probe: How many employees are currently working at the company?

Probe: Do you consider the size of the company to be small, medium, or large?

Probe: What makes the company unique in the natural health products industry?

1. Can you tell me a little bit about your educational or professional background?
2. What, if any, changes have been made to how the company operates since the implementation of the Natural Health Products Regulations? Can you provide an example?

Probe: Did you need to comply with GMPs?

Probe: Who has implemented these changes at the company?

Probe: Have you had to hire any new personnel or train employees in order to comply with the Regulations? Can you provide an example?

1. What strategies or tactics has your company employed to deal with the changes brought on by the Regulations?
2. What have been the major challenges in attempting to be compliant with the Regulations?
3. Has your company submitted a Natural Product Licence (NPN) application to the Natural Health Products Directorate (NHPD)? If so, please complete Form A.

Probe: How many NPN applications have you submitted?

Probe: How many NPN applications do you intend on submitting?

**If yes, proceed with following questions:**

1. Why was an NPN application submitted for these products and not other NHPs? Was there some sort of strategy?
2. Describe your experiences with being involved in submitting a natural product licence (NPN) application to the Natural Health Products Directorate (NHPD).

Probe: How long did this process take?

Probe: Was it clear what you needed to do?

1. What has been the result of the application?
2. What are your major areas of concern for submitting these applications?
3. Are there any differences in completing an application for a compendium, traditional, or nontraditional product?

Probe: Does the amount of time needed to complete the different types of forms vary?

Probe: Do more people at the company need to be involved depending on the application type?

**If no, proceed with the following questions**

1. Why hasn’t your company submitted an NPN application, to date?

Probe: Does your company intend on submitting NPN applications in the future? Why or why not?

Probe: How many applications will be submitted to the NHPD?

1. Has the NHPD applied any enforcement actions to the company?

**Questions to be asked after either of the responses to Question #6:**

1. Has the NHPD provided any information, assistance, or feedback to your company to help you become compliant with the new regulations? Can you provide an example?
2. What suggestions do you have for the NHPD to help industry members with these Regulations?
3. Are you satisfied with the final version of the NHP regulations? Why or why not?
4. Why do you think these Regulations were implemented?

Probe: Who pushed for these Regulations to move forward?

**University of Toronto**


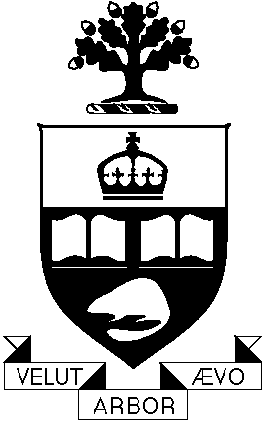


**Form 1: Participant Questionnaire**

| **Name:** |  |
| --- | --- |
| **Company Name:** |  |
| **Position/Title:** |  |
| **Number of Years Working at this Company:** |  |
| **List of Main Responsibilities and Description:** |  |

**University of Toronto**


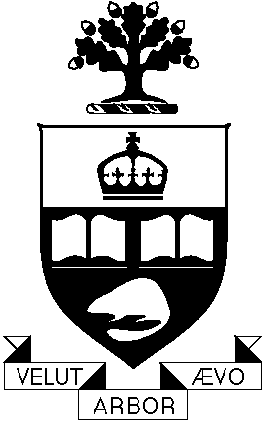


**Form 2: Company Characteristics**

| Key Characteristics of the Company | |
| --- | --- |
| Number of Employees [19] | 1 – 4  5 – 9  10 – 19  20 – 49  50 – 99  100 – 199  200 – 499  500 – 999  1000 + |
| Number of products sold |  |
| Number of NHP products sold |  |
| First date of company operations |  |
| *Date the company entered the NHP market* |  |
| Growth stage of the Company  (start-up, low growth, high growth, mature) |  |
| # of products currently having a DIN number |  |
| Market focus  (local, regional, national, international) |  |
| Total Sales (Canadian $) [19] | N/A  < $100,000  $100,000 - 199,999  $200,000 - 499,999  $500,000 - 999,999  $1,000,000 - 4,999,999  $5,000,000 - 9,999,999  $10,000,000 - 24,999,999  $25,000,000 - 49,999,999  $50,000,000 + |

**University of Toronto**


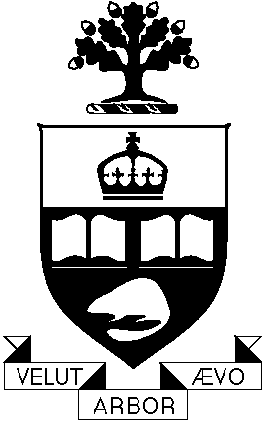


**Form 3: Questionnaire for Companies that Submitted NPN Applications for Glucosamine and Chondroitin**

|  | **Brand Name** | **Other Active Ingredients** | **Date Submitted** |
| --- | --- | --- | --- |
| **Submitted NPN applications for Chondroitin** |  |  |  |
| **Total Number** |  |  |  |
| **Submitted NPN applications for Glucosamine** |  |  |  |
| **Total Number** |  |  |  |
| **Submitted NPN applications for combination products (glucosamine and chondroitin)** |  |  |  |
| **Total Number** |  |  |  |
| **Grand Total** |  |  |  |
